# Accuracy of hyaluronic acid level for predicting liver fibrosis stages in patients with hepatitis C virus

**DOI:** 10.1186/1476-5926-4-6

**Published:** 2005-07-11

**Authors:** Philippe Halfon, Marc Bourlière, Guillaume Pénaranda, Romaric Deydier, Christophe Renou, Danielle Botta-Fridlund, Albert Tran, Isabelle Portal, Isabelle Allemand, Alessandra Rosenthal-Allieri, Denis Ouzan

**Affiliations:** 1 Department of virology, Alphabio Laboratory, Marseille, France; 2 Department of Hepato-Gastroenterology, Saint-Joseh Hospital, Marseille, France; 3 Department of Hepato-Gastroenterology, Hyères Hospital, Hyères, France; 4 Department of Hepato-Gastroenterology, La Conception Hospital, Marseille, France; 5 Department of Hepato-Gastroenterology, Archet Hospital, Nice, France; 6 Department of Hepato-Gastroenterology, Arnault Tzanck Institute, Saint Laurent du Var, France

## Abstract

**Background:**

In patients with chronic hepatitis C virus, liver biopsy is the gold standard for assessing liver disease stage; nevertheless, it is prone to complications, some of them serious. Non-invasive methods have been proposed as surrogate markers for liver fibrosis. It was shown that serum hyaluronic acid (HA) level increases with the development for liver fibrosis. The aim of this study was to evaluate the diagnostic value of HA as well as to determine the HA level cut-off for predicting the presence or absence of fibrosis, severe fibrosis, and cirrhosis.

**Results:**

405 patients with chronic hepatitis C were prospectively included with biomarker measurement and liver biopsy done the same day: 151 in the training set (only biopsy lengths of 25 mm or more) and 254 in the validation set. For the discrimination of significant fibrosis, severe fibrosis, and cirrhosis in the training set, the areas under curve (AUCs) were 0.75 ± 0.03, 0.82 ± 0.02, and 0.89 ± 0.03, respectively. Absence of significant fibrosis, severe fibrosis, and cirrhosis can be predicted by HA levels of 16, 25, and 50 μg/l, respectively (with negative predictive values of 82%, 89%, and 100%, in the same order). Presence of significant fibrosis, severe fibrosis, and cirrhosis can be predicted by HA levels of 121, 160, and 237 μg/l, respectively (with positive predictive values of 94%, 100%, and 57%, in the same order).

**Conclusion:**

In the validation set, HA was accurate in predicting significant fibrosis, severe fibrosis, and cirrhosis with AUCs of 0.73, 0.77, and 0.97, respectively. Moreover, accurate HA level cut-offs were defined for predicting significant fibrosis, severe fibrosis, and cirrhosis. Thus, the study supports that HA level may be clinically useful as a non-invasive marker for liver fibrosis and/or cirrhosis.

## Background

Liver biopsy is currently recommended as the gold standard method of staging fibrosis in patients with chronic hepatitis C [1,2]. The risk of developing cirrhosis depends on the stage (degree of fibrosis) and the grade (degree of inflammation and necrosis) observed in the initial liver biopsy [3,4]. This procedure, however, is invasive and has potential complications [5,6]. Non-invasive approaches developed to assess histological samples include clinical symptoms, routine laboratory tests, and radiolographic imaging [7-10]. Several clinical studies have attempted to identify serum markers that correlate with the degree of fibrosis and thus could be used, with feasibility, in conjunction with or in place of a liver biopsy [2-4,6,8,9]. The serum markers of fibrogenesis include platelet count [11], prothrombin time [12], the ratio of alanine aminotransferase and aspartate aminotransferase levels [8], gamma-glutamyl transferase level [13], and serum albumin level [14]. Fibrotest (FT) is a simple non-invasive panel of biochemical markers for fibrosis and activity [15].

Another non-invasive approach relies on the measurement of substances that regulate fibrosis or participate in the generation of the liver extracellular matrix. The most applicable include hyaluronic acid (HA) [16], type IV collagen [17], N-terminal propeptide of type III procollagen [18], metalloproteinases [19], inhibitors of metalloproteinases [19], and growth-transforming factor beta [20]. HA is a high molecular weight glycosaminoglycan, which is an essential component of extracellular matrix in virtually every tissue in the body [21]. In the liver, HA is mostly synthesized by the hepatic stellate cells and degraded by the sinusoidal endothelial cells [6]. HA levels are increased in chronic liver diseases [6]. In patients with chronic hepatitis C virus (HCV), HA levels increase with the development of liver fibrosis. Moreover, in patients with cirrhosis, HA levels correlate with clinical severity [7,10,11].

The first aim of this study was to evaluate the diagnostic value of HA for significant fibrosis (F2-F4), severe fibrosis, (F3F4) and cirrhosis (F4), in patients with HCV infection. The second aim was to determine the serum HA level cut-off to predict both presence and absence of F2-F4, F3F4, and F4.

## Results

### Patients

The cohort included 405 patients. Table 1 shows the patient characteristics at the time of liver biopsy. The training and validation sets did not significantly differ in any of the assessed variables. Of the patients, 47% (190/405) had significant fibrosis (F2-F4), 24% (99/405) had severe fibrosis (F3F4), and 6% (25/405) had cirrhosis.

**Table 1:** Characteristics of the 405 patients at the time of liver biopsy (comparison between the training and the validation sets).

**Characteristics**	**Training set (n = 151)**	**Validation set (n = 254)**	**All patients (n = 405)**
Age (Mean ± SD)	51 ± 14	47 ± 12	49 ± 13
Male (n (%))	82 (54)	133 (52)	215 (53)
HA (μg/l) (Mean {95% CI})	63 {47;79}	53 {41;65}	57 {47;67}
Stage of fibrosis (n (%))			
0	28 (19)	33 (13)	61 (15)
1	51 (34)	103 (41)	154 (38)
2	33 (22)	58 (23)	91 (23)
3	27 (18)	47 (18)	74 (18)
4	12 (7)	13 (5)	25 (6)

### Hyaluronic acid and fibrosis in the training set

**Figure 1:**
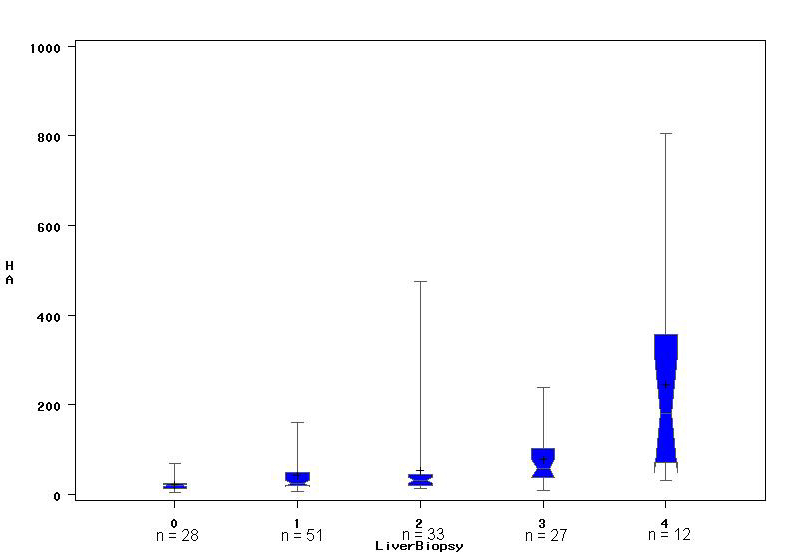
Box & Whisker plot representing the relation between the stage of fibrosis and HA level. The line through the box is the median; the top and bottom edges of each box represent the 25^th ^and 75^th ^percentiles, giving the interquartile range; and the cross in the box is the mean. The vertical lines at each side of the box represent distribution from the quartile to the farthest observation. The curve represents the HA median value of each fibrosis stage (F0: 20 μg/l, F1: 25 μg/l, F2: 30 μg/l, F3: 58 μg/l, and F4: 180 μg/l). The relation between the stages of fibrosis and HA level was statistically significant (Kruskal-Wallis – *p *< 0.0001). Spearman rank correlation coefficient (r) between the stage of fibrosis and HA level was 0.55.

Figure 1 shows HA levels and stages of fibrosis are well correlated (Spearman r = 0.55 – p < .0001). Although there is an overlap between HA levels and fibrosis determined by liver biopsy, there is a significant increase in HA levels when considering F0 to F4 scores (Kruskal-Wallis – *p *< 0.0001).

### Fibrosis, severe fibrosis, and cirrhosis diagnosis

**Figure 2:**
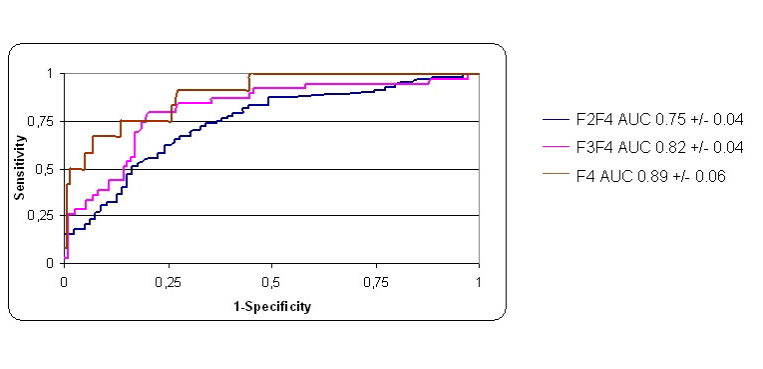
Receiver operating characteristic curves of HA for the prediction of significant fibrosis (F2-F4), severe fibrosis (F3F4), and cirrhosis (F4) in the training set.

**Figure 3:**
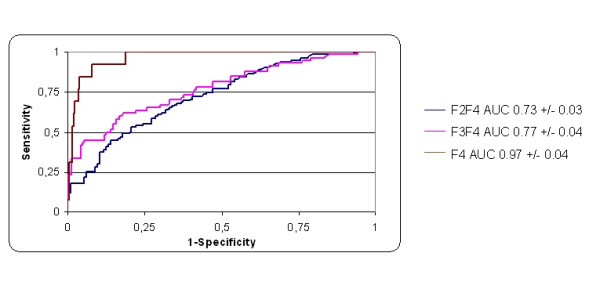
Receiver operating characteristic curves of HA for the prediction of significant fibrosis (F2-F4), severe fibrosis (F3F4), and cirrhosis (F4) in the validation set.

Figure 2 and 3 shows receiver operating characteristic curves of discriminatory values of HA according to the severity of liver fibrosis in the training and validation sets. For the discrimination of fibrosis, severe fibrosis, and cirrhosis in the training set, areas under curve (AUCs) were (Mean ± SE) 0.75 ± 0.03, 0.82 ± 0.02, and 0.89 ± 0.03, respectively. In the validation set, AUCs were 0.73 ± 0.03, 0.77 ± 0.04, and 0.97 ± 0.04, respectively.

#### Fibrosis

Two cut-off values were chosen for identifying absence (less than 16 μg/l) and presence (greater than 121 μg/l) of significant fibrosis (F2-F4). Applying the lower cut-off, the presence of significant fibrosis could be excluded with a high certainty as only 3 (17%) of the 18 patients with an HA level below 16 μg/l had significant fibrosis, with a negative predictive value (NPV) of 83%. In the validation set, 11 (18%) of 60 patients with a score below 16 μg/l had significant fibrosis (NPV of 82%). Applying the high cut-off to the training group (HA greater than 121 μg/l), only 2 (13%) of the 15 patients with HA greater than 121 μg/l had no fibrosis, with a positive predictive value (PPV) of 87%. In the validation set, 16 of the 17 patients with HA greater than 121 μg/l had significant fibrosis (PPV of 94%) (Table 2).

**Table 2:** Diagnostic performance of HA in the validation set.

**HA cut-off**	**Sensitivity (%)**	**Specificity (%)**	**NPV (%)**	**PPV (%)**	**Population involved (%)**	**Interpretation**
<16	91	36	82	55	24	Absence of fibrosis (F0F1) (82% certainty)
>121	14	99	57	94	7	Presence of fibrosis (F2) (94% certainty)
≤ 25	78	53	89	34	46	Absence of severe fibrosis (F0F1F2) (89% certainty)
>160	22	100	81	100	5	Presence of severe fibrosis (F3) (100% certainty)
≤ 50	100	79	100	20	75	Absence of cirrhosis (F0F1F2F3) (100% certainty)
>237	31	99	96	57	3	Presence of cirrhosis (F4) (57% certainty)

Note 1: Accuracy = Sensitivity + Specificity + NPV + PPV. Note 2: Population involved stands for the proportion of patients who fall in the corresponding cut-off.

#### Severe fibrosis

As for significant fibrosis diagnosis, 2 cut-off values were chosen for identifying absence (less than 25 μg/l) and presence (greater than 160 μg/l) of severe fibrosis (F3F4). Applying the lower cut-off, only 3 (5%) of the 64 patients with HA lower than 25 μg/l had severe fibrosis (NPV of 95%). In the validation set, only 13 (11%) of the 123 patients with HA lower than 25 μg/l had severe fibrosis (NPV of 89%). Applying the higher cut-off (HA greater than 160 μg/l) to the training set, 10 (NPV of 91%) out of 11 patients with HA greater than 160 μg/l had severe fibrosis, and none of the 13 patients with HA greater than 160 μg/l from the validation set had severe fibrosis (PPV of 100%) (Table 2).

#### Cirrhosis

Two cut-off values were chosen for identifying absence (less than 50 μg/l) and presence (greater than 237 μg/l) of cirrhosis (F4). Applying the lower cut-off, 100 (NPV of 99%) of the 101 patients with HA lower than 264 μg/l had no cirrhosis. In the validation set, none of the patients with HA lower than 50 μg/l had cirrhosis (NPV of 100%). When applying the higher cut-off (237 μg/l) to the training set, a fair PPV of 71% was obtained for predicting the presence of cirrhosis. The PPV was lower when applying the cut-off to the validation set (PPV of 57%) (Table 2).

## Discussion

Combining HA level with other serum markers for assessing liver fibrosis has been considered in some other studies [22,23]. One of the interests of our study was to focus on the diagnostic accuracy of HA alone in predicting fibrosis and cirrhosis in HCV-infected patients.

In the present study, HA level was accurate in predicting significant fibrosis, severe fibrosis, and cirrhosis, with AUCs of 0.75, 0.82, and 0.89, respectively, in the training set; and of 0.73, 0.77, and 0.97, respectively, in the validation set. Using values below the lower cut-off level or above the higher cut-off level, one could predict absence or presence of significant fibrosis, severe fibrosis, and cirrhosis in 31%, 51%, and 78%, respectively, in the validation set patients. Therefore, it is likely that the association of HA level with the degree of hepatic fibrosis represents an indirect one, expressing the functional correlation between fibrosis and both the concomitant capillarization and hepatic hemodynamic changes.

Significant fibrosis can be predicted by a HA level of <16 μg/l for its absence (NPV of 82%) and of >121 μg/l for its presence (PPV of 94%) in the validation set. Severe fibrosis can be predicted by a HA level of ≤ 25 μg/l for its absence (NPV of 89%) and of >160 μg/l for its presence (PPV of 100%). Cirrhosis can be predicted by an HA level of ≤ 50 μg/l for its absence (NPV of 100%) and of > 237 μg/l for its presence (PPV of 57%). Considering a serum HA cut-off of 60 μg/l for absence of cirrhosis diagnosis, our data are in the same range as those of other studies [3,24].

A cut-off value of 110 μg/l was suggested for separating patients with and without cirrhosis [18]. Taking in consideration this cut-off in our cohort of patients, a misclassification of cirrhotic patients was observed in 23% (3/13) (sensitivity of 77%) of patients with proven cirrhosis. This difference may be due to the use of a different HA assay.

Our study included a sufficient proportion of patients with significant fibrosis: 47% in the training set and 46% in the validation set; however, the proportion of patients with cirrhosis was low in the two sets: 7% and 5%, respectively. The second limitation of this study is the number of unclassified patients (between 22% and 69%).

## Conclusion

This study showed that significant fibrosis, severe fibrosis, and cirrhosis can be predicted by serum HA levels in patients with HCV infection. The notion of routinely measuring a marker that reflects the function of the sinusoidal endothelial cells, rather than the hepatocytes themselves, is an exciting concept. Serum HA would be clinically useful as a non-invasive marker of liver fibrosis or cirrhosis in HCV-infected patients. It suffers from the need to limit, as much as possible, potential confounding variables such as the effects of exercise and eating.

Further studies conducted in a large cohort of cirrhosis patients are needed to corroborate this study, namely because few of our patients had cirrhosis and the cut-off levels must be considered in an independent study. Moreover, a comparison of HA levels with other non-invasive markers and scores of liver fibrosis (FT, APRI, Forns, age-platelets index, platelet count, prothrombin time, etc.) would be of interest.

## Methods

### Study participants

The cohort included 405 patients (mean age 49 ± 13 years, 53% men) with HCV infection between November 2002 and December 2003, in five centers in southern France [Conception Hospital and Saint-Joseph Hospital (Marseille), Archet Hospital (Nice), Hyères Hospital (Hyères), and Arnault Tzanck Institute (St Laurent du Var)]. HA assessment was evaluated with data from 151 patients (training group) and was validated in the remaining 254 patients (validation group). Only biopsy lengths of 25 mm or more were included in the training group, in accordance with a recent study [25]; the remaining patients were included in the validation set. None of the patients had joint injuries, based on clinical examinations and inflammatory markers estimated at the time of inclusion in the study. None of the patients had renal impairment, based on normal creatinine clearance (Cockroft calculation).

### Liver biopsies

Liver biopsy examination was performed in each center by evaluating the stage of fibrosis and grade of activity according to the METAVIR scoring system [26,27]. Liver biopsies were histologically assessed in the local centers and a second assessment of each biopsy was done in a reference laboratory. The second assessments were done by the same pathologist. In this study, all liver biopsies were re-staged by the central reference laboratory (n = 405). No liver biopsies were found with more than one-stage difference between the local pathologist and the reference pathologist. One-stage discordance is considered pathology-dependent and thus not considered significant. Fibrosis was staged on a scale of 0 to 4: F0 = no fibrosis, F1 = portal fibrosis without septa, F2 = portal fibrosis and few septa, F3 = numerous septa without cirrhosis, F4 = cirrhosis.

### Hyaluronic acid

HA levels were measured by Alphabio Laboratories, Marseille, with the Corgenix Hyaluronic Acid Test Kit, Corgenix Inc., CO, following the manufacturer's instructions (patients in fasting conditions, no physical effort). Each HA level was measured in duplicate (range 1 to 871 μg/l) and a pool control set was used. All serum samples were obtained on the day of liver biopsy.

### Statistical analysis

The association between HA levels and liver biopsy staging was measured with the Kruskal-Wallis multiple comparison test and with the Spearman rank correlation coefficient. A *p *< 0.05 was considered significant. Respective diagnostic values were reported by the area under the receiver operating characteristic curves (AUC) (± standard error mean), NPV, PPV, sensitivity, and specificity.

## Authors' contributions

PH and MB conceived and wrote the manuscript. RD, CR, DBF, AT, IP, IA, ARA and DO were responsible for the patient drafting, carried out biochemical analysis, participated in the coordination of the study, and drafted the paper. GP performed the statistical analysis and participated in the writing of the paper. All authors read and approved the final manuscript
